# Quantum magnetic phase transition in square-octagon lattice

**DOI:** 10.1038/srep06918

**Published:** 2014-11-05

**Authors:** An Bao, Hong-Shuai Tao, Hai-Di Liu, XiaoZhong Zhang, Wu-Ming Liu

**Affiliations:** 1 Laboratory of Advanced Materials, Department of Materials Science and Engineering, Tsinghua University, Beijing 100084, China; 2 School of Mathematics, Physics and Biological Engineering, Inner Mongolia University of Science and Technology, Baotou 014010, China; 3 Beijing National Laboratory for Condensed Matter Physics, Institute of Physics, Chinese Academy of Sciences, Beijing 100190, China

## Abstract

Quantum magnetic phase transition in square-octagon lattice was investigated by cellular dynamical mean field theory combining with continuous time quantum Monte Carlo algorithm. Based on the systematic calculation on the density of states, the double occupancy and the Fermi surface evolution of square-octagon lattice, we presented the phase diagrams of this splendid many particle system. The competition between the temperature and the on-site repulsive interaction in the isotropic square-octagon lattice has shown that both antiferromagnetic and paramagnetic order can be found not only in the metal phase, but also in the insulating phase. Antiferromagnetic metal phase disappeared in the phase diagram that consists of the anisotropic parameter *λ* and the on-site repulsive interaction U while the other phases still can be detected at T = 0.17. The results found in this work may contribute to understand well the properties of some consuming systems that have square-octagon structure, quasi square-octagon structure, such as ZnO.

The discovery and classification of quantum phases of matters and the transition between these distinctive phases have been recurring theme in condensed matter physics for many years and still wheel the researchers' extensive interests^1^,^2^,^3^,^4^,^5^,^6^,^7^,^8^,^9^,^10^,^11^,^12^,^13^,^14^,^15^. Notable quantum phases, such as super-conductivity, quantum hall effect, Mott insulating phase and topological phase, have great significance in theoretical investigations and promising potential in applications. These exotic phases have been found in many quantum systems with quite common structure, such as the honeycomb lattice, the triangular lattice, the decorated honeycomb lattice, the kagomé lattice and so forth^16^,^17^,^18^,^19^,^20^,^21^,^22^,^23^,^24^,^25^,^26^,^27^,^28^,^29^,^30^,^31^. Recently a unique quantum many particle lattice system named square-octagon lattice have been investigated in theoretical way intensively and a plenty of meaningful results have been presented. Researchers have found topological phases and the transitions between these novel phases in the square-octagon lattice that 1/4 and 3/4 filled with fermions under the framework of the tight binding model through considering the spin-orbit coupling fermions^32^. Another one theoretical joy models named Fully packed loop model also has been adopted to investigate the square-octagon lattice^33^. Additionally, researchers have found quasi square-octagon structure in (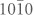) surface of functional material ZnO by first principle calculations and aberration-corrected transmission electron microscopy (ACTEM) observation experimentally during its pressure induced phase transition process^34^.

However, few of the previous work related to the square-octagon lattice considered the particles' on-site repulsive interactions that have crucial effect on the properties of the systems. Therefore in this work, the celebrated Hubbard model^35^,^36^ was used to describe this strongly correlated many particle systems for the purpose of understanding well the influences of interaction on the properties of the square-octagon lattice with fermions. The cellular dynamical mean field theory (CDMFT)^37^,^38^,^39^,^40^,^41^,^42^, which maps the lattice to a self-consistent embedded cluster in real space, was adopted to deal with the Hubbard model and the continuous time quantum Monte Carlo (CTQMC)^47^ algorithm was used as a impurity solver to deal with the mean field equations. The CDMFT is proved to be more successful than the dynamical mean field theory and the CTQMC is more accurate than the general quantum Monte Carlo method. Based on the single-particle Greens function given by the CDMFT and CTQMC, the single-particle density of states and the double occupancy which play critical role in the identification of Mott metal-insulator transition have been calculated. The phase diagram which composed of the on-site interaction and the energy gap, the relationship between the interaction and magnetic properties of the systems also have investigated through defining the magnetic order parameter. We also presented phase diagram which consists of the competition between the temperature and on-site repulsive interaction for isotropic square-octagon lattice and the the competition between the anisotropy and on-site repulsive interaction.

## Results

### Strongly correlated square-octagon lattice system

**Figure 1:**
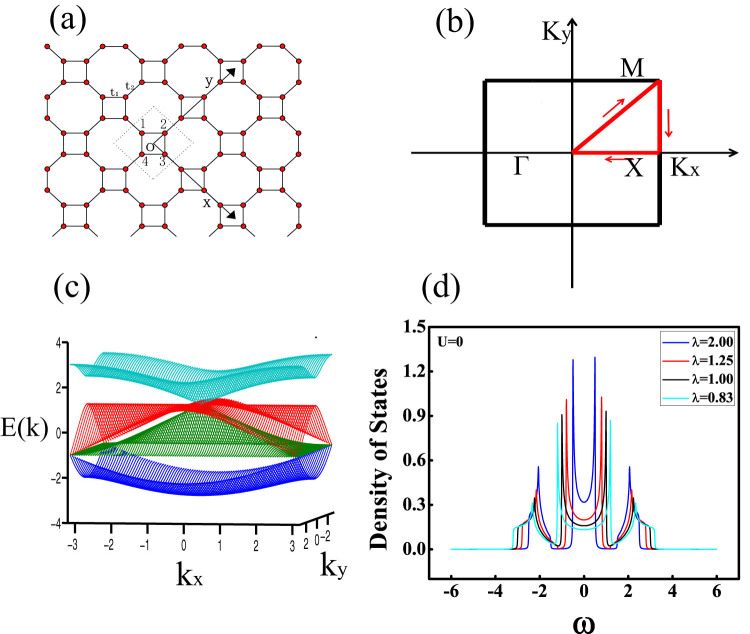
Sketch of square-octagon lattice. (a) Sketch of the square-octagon lattice and illustration of the nearest-neighbor hopping t_1_ and t_2_, where t_1_ and t_2_ represents the hopping factors between the nearest-neighboring sites in the same square and between the endpoints of the different squares' linking line, respectively. (b) Structure of the first Brillouin zone of square-octagon lattice. (c) Energy band in the first Brillouin zone of the square-octagon lattice. (d) Density of states of the square-octagon lattice without interaction for different anisotropic parameter *λ* while T = 0.2.

The square-octagon lattice is a bipartite lattice that can be thought of as a square lattice in which each vertex has been decorated with a tilted square, as shown in Fig. 1 (a) and its first Brillouin zone in Fig. 1 (b). It has the same coordination number as the honeycomb lattice has and its boundary shapes armchair. It enjoys the symmetry of the square lattice and symmetrically it satisfies C_4_ point group.

The standard Hubbard model is adopted to investigate the square-octagon lattice and the Hamiltonian can be written as follows, 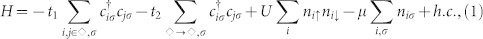
where 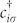 and *c_iσ_* represent creation and annihilation operator of fermions with spin *σ* on site i respectively, while 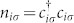 denote the particle number operator on lattice site i. The value of spin index *σ* is spin up or spin down. The first two terms in this Hamiltonian account for the kinetic energy of the system, which is characterized by the coefficient factor t_1_ and t_2_. t_1_ represents the hopping between the nearest neighboring sites in the same square lattice and t_2_ is the hopping between the endpoints of the liking line of the two nearest neighboring square lattice. The third term describes the on-site repulsive interaction (U > 0) between fermions with opposite spin. Here we set *t*_1_ as energy unit (*t*_1_ = 1). *μ* is chemical potential and in order to reach half filled case *μ* should equals zero for this lattice system. We also defined an anisotropic parameter *λ* which equals to the ration *t*_1_/*t*_2_ (*λ* = *t*_1_/*t*_2_).

For the case of U = 0 and *μ* = 0, the Hubbard model transmits to the tight binding model and the Hamiltonian in the momentum space is 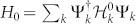, in which Ψ*_k_* = (*c*_1*k*↑_, *c*_2*k*↑_, *c*_3*k*↑_, *c*_4*k*↑_, *c*_1*k*↓_, *c*_2*k*↓_, *c*_3*k*↓_, *c*_4*k*↓_)^T^. The index i = 1, 2, 3,4 in creation and annihilation operators represent the four sites in each unit cell as illustrated in Fig. 1 (a) and k is the locations in the first Brillouin zone. ↑ and ↓ hint the spin-up and spin-down states respectively. 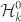 takes the following form 
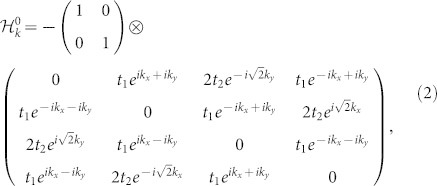


Since *H*_0_ is decoupled in spin states, so 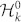 is block diagonalized, i.e. two blocks representing spin-up and spin-down electrons are the same. The energy band of the first Brillouin zone of the square-lattice under the frame of the tight biding model has been obtained through diagonalizing 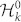 and shown in Fig. 1 (c). The density of states of the square-octagon lattice half filled with fermions without interaction at T = 0.2 for different anisotropic parameter *λ* in Fig. 1 (d).

**Figure 2:**
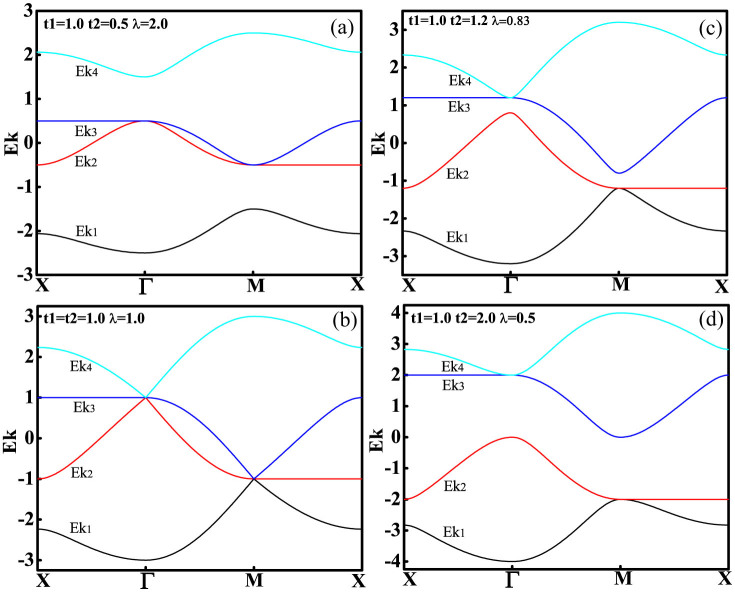
Energy band of isotropic square-octagon lattice along the line between high symmetric points in the first Brillouin zone. *t*_1_ fixed for all case and equals 1. (a) The band Ek_2_ and Ek_3_ contact each other at Γ point and M point for *λ* = 2.0 and the system is in metallic state. (b) Energy band Ek_2_, Ek_3_ and Ek_4_ cross at Γ point while Ek_1_, Ek_2_ and Ek_3_ cross at M point for *λ* = 1. (c) The band Ek_1_ and Ek_2_ contact at M point while the band Ek_3_ and Ek_4_ contact at Γ point for *λ* = 0.83 and the system is still in metallic states. (d) The band Ek_2_ and the band Ek_3_ are completely separated by Fermi energy level and the system turns into insulating states.

In order to get the effect of anisotropic parameter *λ* and the value of hopping term *t*_1_ and *t*_2_ on the phase transitions, we presented the energy band along the line between the high symmetric points in the first Brillouin zone in Fig. 2 even has shown the 3-dimensional energy band in the first Brillouin zone in Fig. 1 (c). The energy band Ek_2_ and Ek_3_ touch at Γ point and M point for *λ* = 2.0 in Fig. 2 (a). Energy band Ek_2_, Ek_3_ and Ek_4_ cross at Γ point while Ek_1_, Ek_2_ and Ek_3_ cross at M point for *λ* = 1, the system is in metallic states. It can be seen that with the decreasing of anisotropic parameter *λ*, Ek_2_ and Ek_3_ separate and meanwhile Ek_1_ and Ek_2_ contact at M point, Ek_3_ and Ek_4_ contact at Γ point while *λ* = 0.83. The system is still in metallic states. As Fig. 2 (d) shows that energy band Ek_2_ and Ek_3_ completely separated by the Fermi energy level while *λ* = 0.5 and the system turns into insulating states.

### Phase diagrams of the square-octagon lattice

**Figure 3:**
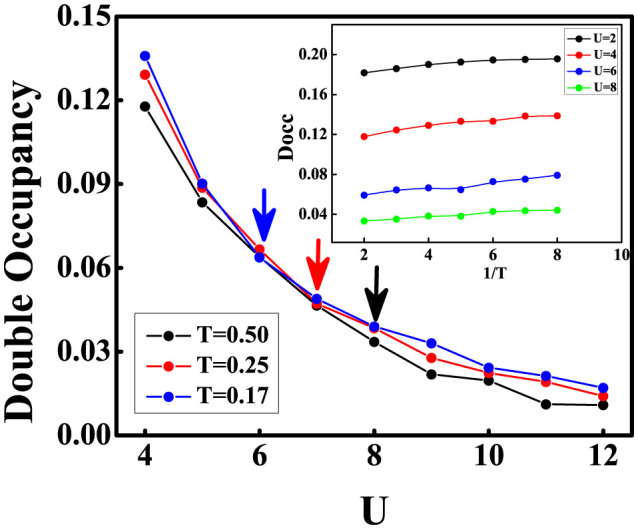
Double occupancy of the square-octagon lattice. (a) The evolution of the double occupancy as a function of on-site repulsive interaction U for different temperature T. The arrows hint the corresponding value of Mott transition for different temperature. (b) is the value of the double occupancy under certain temperature for different on-site repulsive interaction U.

With the increase of on-site repulsive interaction U the the probability of more than one fermions occupying the same lattice site will reduce and eventually only one fermion confined in per lattice site at certain large value of U. The confinement of fermion in one lattice site is described by double occupancy (Docc)^43^ which is an important quantity that used to characterize the critical point in Mott phase transitions and indicates the transition order, and also can be used to describe the localization of the electrons in strongly correlated electron systems. The formula of double occupancy is 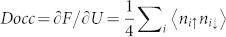, where *F* is free energy. The double occupancy of isotropic square-octagon lattice as a function of interaction for fixed temperature and as a function of temperature for fixed interaction have been shown in outer part and inner part of Fig. 3 respectively. It can be seen in the outer part of Fig. 3 that Docc decreases as the interaction increases due to the suppressing of the itinerancy of the atoms. When the interaction is stronger than the critical interaction of the Mott transition, the effect of the temperature on Docc is weakened and Docc for different temperatures consistently trend to zero, which shows the temperature does not affect the double occupancy distinctly. The continuity of the evolution of the double occupancy by interaction shows that it is a second-order transition. We also have shown the relation between Docc and the temperature at different interaction in inner part of Fig. 3. From the inner part of Fig. 3 we can find that the double occupancy decreases with the increase of the temperature for fixed on-site repulsive interaction.

**Figure 4:**
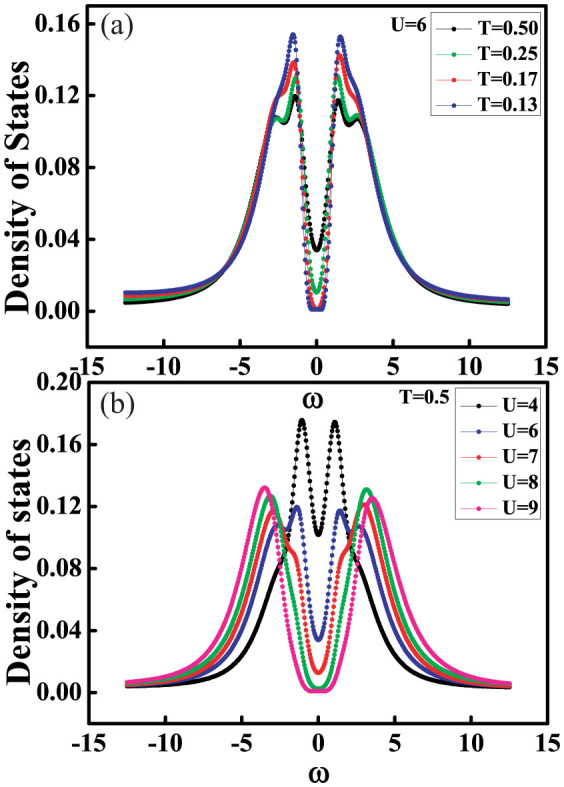
Density of states of the isotropic square-octagon lattice. (a) depicts the density of states for the different temperature T while the interaction fixed at *U*/*t*_1_ = 6. (b) shows the density of states for different interaction U while the temperature fixed *T*/*t*_1_ = 0.5.

The density of states is one of the most important quantities in the characterization of the Mott metal-insulator phase transition of Hubbard model. For the purpose of investigating the Mott metal-insulator phase transition as the evolution of single particle spectral^44^, we defined Density of states, the formula is 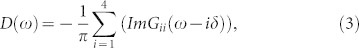
where *i* is the lattice points index in the cluster. The Density of states can be derived from the imaginary time Greens function *G*(*τ*) by using the maximum entropy method^45^. Fig. 4 (a) and (b) respectively shows the density of states of isotropic square-octagon lattice for different temperature at *U*/*t*_1_ = 6 and the density of states for different repulsive interactions while *T*/*t*_1_ = 0.5. The inner part of Fig. 4 (a) is the density of states of system for *U*/*t*_1_ = 0 and *T*/*t*_1_ = 0.17. It can be evidently seen in Fig. 3 that the systems will change from metal state to Mott insulating state which characterized by the opened gap at *ω* = 0 with the increase of the repulsive interaction for fixed temperature and the decrease of the temperature for the fixed repulsive interactions. However, the evolution shape of the density of states with the change of frequency in this two cases is much different from each other. The critical point between paramagnetic metal state and Mott insulating state is (*T*/*t*_1_ = 0.17, *U*/*t*_1_ = 6), (*T*/*t*_1_ = 0.25, *U*/*t*_1_ = 7) and (*T*/*t*_1_ = 0.5, *U*/*t*_1_ = 8).

**Figure 5:**
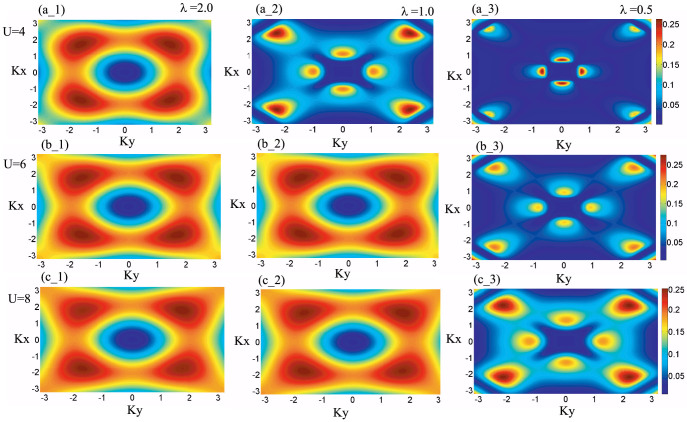
Fermi surface evolution of the square-octagon lattice. pictures in the every same row depict the Fermi surface evolution for fixed interaction and different anisotropic parameters while the pictures in the same column shows the Fermi surface evolution for fixed anisotropic and different interaction. Peaks in the diagrams represent the dominant spectral weight of electrons with zero energy in momentum space and thus correspond to the location of Fermi surface. With the increase of U and *λ*, the renormalization effect becomes stronger and the distribution spread. Fermi surface evolution is obtained at temperature *T*/*t*_1_ = 0.1.

In order to describe the Fermi surface evolution, we defined the spectral function 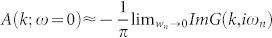. A linear extrapolation of the first two Matsubara frequencies is used to estimate the self-energy to zero frequency. The Fermi surface of isotropic square-octagon lattice half filled with fermions for different interaction *U*/*t*_1_ at fixed temperature *T*/*t*_1_ = 0.1 is shown in Fig. 5 (a_ 2), (b_ 2) and (c_ 2). We also have shown the Fermi surface of anisotropic square-octagon lattice in Fig. 5 for *U*/*t*_1_ = 4, 6, 8 while *T*/*t*_1_ = 0.1. With the decreasing of the *λ* for fixed interaction the amplitude of the spectral weight becomes bigger due to the localization of particles.

**Figure 6:**
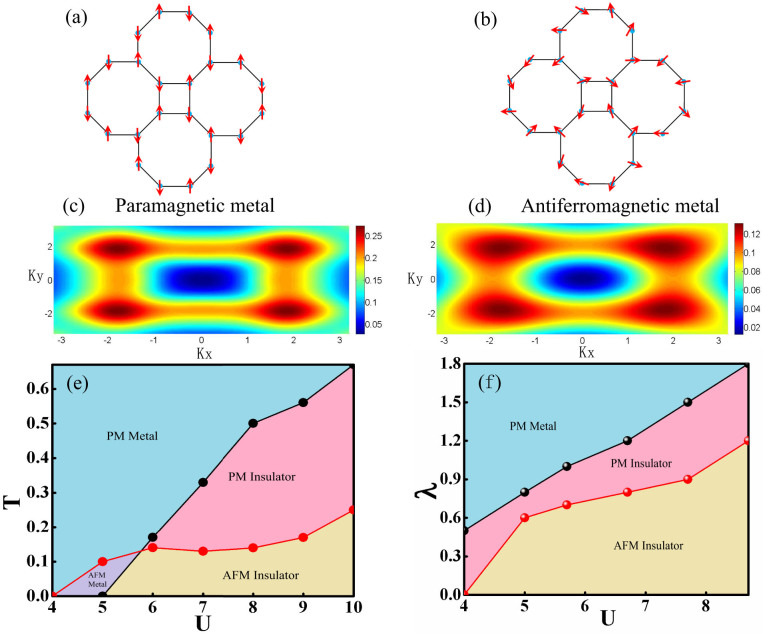
Phase diagrams of the square-octagon lattice. (a) is the sketch of the anti-ferromagnetic order and (b) is for paramagnetic order of square-octagon lattice. (c) Fermi surface evolution of isotropic square-octagon lattice in paramagnetic metal state for T = 0.17. (d) Fermi surface evolution of isotropic square octagon lattice in antiferromagnetic metal state for T = 0.17. (e) Competition between the temperature T and on-site repulsive interaction U for *λ* = 1. The black line is the boundary between metal phase and insulator phase while the red one distinguishes the paramagnetic (PM) phase and anti-ferromagnetic (AFM) phase. (f) *λ* - U phase diagrams of the square-octagon lattice for T = 0.17. The anti-ferromagnetic metal phase will disappear at the high enough temperature while the rest of phases still can be found.

**Figure 7:**
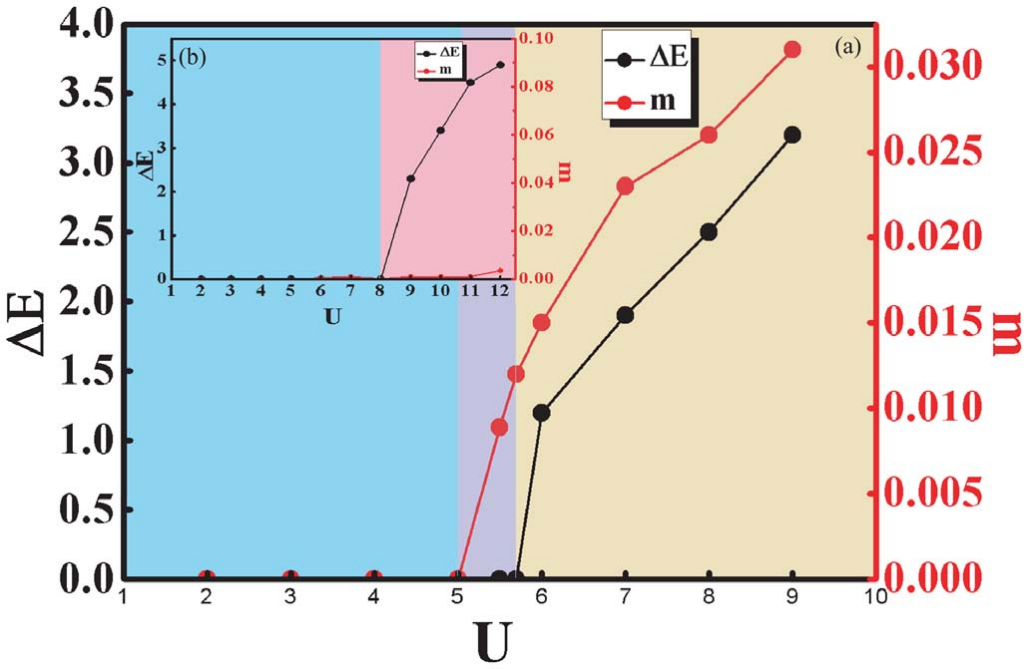
Relationship between energy gap and the on-site repulsive interaction. (a) depicts the relationship between energy gap and interaction for T = 0.1 while (b) for T = 0.5. For T = 0.1, antiferromagnetic state appears before the appearance of the insulating state while the antiferromagnetic state disappear for T = 0.5. The right vertical axes in (a) and (b) shows the magnetic properties of each state.

Based on the systematic calculations on the quantities mentioned above, we have presented the T - U phase diagram of isotropic square-octagon lattice and the competition between anisotropic parameter *λ* and the on-site repulsive interaction (U) for fixed low temperature *T*/*t*_1_ = 0.17. We also studied the magnetic properties of each phase in the square-octagon lattice by using the magnetic order parameter 
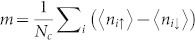
, where 〈*n_iσ_*〉 is the electron density at lattice site *i* with spin index *σ* and sign(i) = 1 if i = 1, 3 and sign(i) = −1 if i = 2, 4 as shown in Fig. 1(a). From the definition of magnetic order it can be known that *m* = 0 correspond to paramagnetic phase while *m* ≠ 0 represents antiferromagnetic phase. Both paramagnetic and anti-ferromagnetic order as shown in Fig. 6 (a) and (b) have been not only found in insulating state but also in the metal state in the T - U phase diagram of isotropic square-octagon lattice. Fermi surface evolution of isotropic square-octagon lattice in paramagnetic metal state in Fig. 6 (c) and in antiferromagnetic metal state in Fig. 6 (d) for for U = 5.5 and T = 0.17. As shown in Fig. 6 (e) that only at low enough temperature or weak enough on-site repulsive scale the systems can transform to antiferromagnetic metal state. The narrow antiferromagnetic metal state region in Fig. 6 (e) means this state is sensitive to the temperature and the on-site repulsive interaction. This results have been confirmed further by the relation between the energy gap and on-site repulsive interaction and the magnetic order parameter m and the on-site repulsive interaction in Fig. 7. The anti-ferromagnetic metal state disappeared in the competition of anisotropic parameter *λ* and interaction diagram while other phases still exist at T = 0.17.

## Discussion

In this work, we use standard Hubbard model to describe the square-octagon lattice and present the quantum magnetic phases and the transition between these novel phases in this many particle systems. We have investigated not only the effect of on-site repulsive interaction of particles with the opposite direction spin on the same site, but also shown the influence of the Kinetic energy of the systems on the phase transitions. We also have studied the magnetic properties of the square-octagon lattice through defining the magnetic order parameter *m*. We hope the results found in this study can be useful for understanding the property of this lattice and the real materials with this structure, even can be helpful for the research on the functional material ZnO with quasi square-octagon lattice.

## Methods

### Cluster dynamical mean-field theory

The cellular dynamical mean-field theory (CDMFT) was used to investigate this many particle square-octagon lattice. In comparison to the general dynamical mean field theory, the cellular dynamical mean field theory gives much more reliable simulation results for low-dimensional system with strong quantum fluctuations due to its efficient consideration of the nonlocal effect. In our case, the cellular dynamical mean field theory maps the original square-octagon lattice onto a 4-site effective cluster embedded in a self-consistent bath field, as shown in Fig. 1 (a). At the beginning of the self consistent calculation process, we guess a mini self-energy Σ(*iω*) which is independent of momentum^46^ and the Weiss field *G*_0_(*iω*) can be obtained by the coarse-grained Dyson equation: 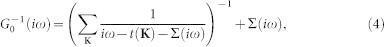
where *ω* is Matsubara frequency, *μ* is the chemical potential, Σ**_K_** is the summation all over the reduced Brillouin zone of the super-lattice. *t*(**K**) is 4 dimensional hopping matrix of super-lattice which drawn from the square-octagon lattice under the framework of cluster dynamical mean field theory.

### Continuous-time quantum Monte-Carlo algorithm

The continuous-time quantum Monte-Carlo (CTQMC) algorithm was used as impurity solver. The CTQMC is based on a series expansion for the partition function in the powers of interaction and the partition function is 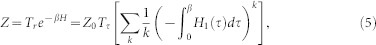
where *T_τ_* is time-ordering operator, 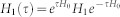 and *H*_1_ is Hamiltonian in interaction picture, 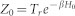 is the partition function for the unperturbed term. Through inserting *H*_1_ = *U*Σ*_i_n_i_*_↑_*n_i_*_↓_ into the partition function and using Wick's theorem further to reform ordering operators in partition functions. The ordering operators can be expressed by the determinants of matrix which consist of the non-interacting Green functions *G*^0^. The new self-energy Σ(*iω*) is recalculated by the Dyson equation: 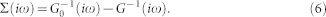


The cluster Green's function *G*(*iω*) can be obtained by CTQMC and 1 × 10^6^ QMC sweeps are carried through for each CDMFT loop^47^. The cluster Green's function both in imaginary time and at Matsubara frequencies: 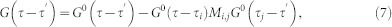

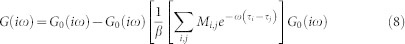
where *G*_0_(*iω*) is a bare Green's function and *M_i_*_,*j*_ is the elements of inverse matrix of matrix that composed of non-interacting Green's functions. The more details about CTQMC can be found in the reference herein^47^.

## Author Contributions

A.B. performed calculations. A.B., H.S.T., H.D.L., X.Z.Z. and W.M.L. analyzed numerical results. A.B., X.Z.Z. and W.M.L. contributed in completing the paper.
